# Comparison of diffusion-weighted MRI and anti-Stokes Raman scattering (CARS) measurements of the inter-compartmental exchange-time of water in expression-controlled aquaporin-4 cells

**DOI:** 10.1038/s41598-018-36264-9

**Published:** 2018-12-18

**Authors:** Takayuki Obata, Jeff Kershaw, Yasuhiko Tachibana, Takayuki Miyauchi, Yoichiro Abe, Sayaka Shibata, Hiroshi Kawaguchi, Yoko Ikoma, Hiroyuki Takuwa, Ichio Aoki, Masato Yasui

**Affiliations:** 1 Applied MRI Research, National Institute of Radiological Sciences, QST, Chiba, 263-8555 Japan; 2 Department of Pharmacology, Keio University School of medicine, Tokyo, 160-0016 Japan; 3 Keio Advanced Research Center for Water Biology and Medicine, Tokyo, 160-0016 Japan; 4 Department of Molecular Imaging and Theranostics, National Institute of Radiological Sciences, QST, Chiba, 263-8555 Japan; 5 Human Informatics Research Institute, National Institute of Advanced Industrial Science and Technology, Tsukuba, 305-8566 Japan; 6 Department of Functional Brain Imaging Research, National Institute of Radiological Sciences, QST, Chiba, 263-8555 Japan

## Abstract

We performed multi-b and multi-diffusion-time diffusion-weighted magnetic resonance imaging on aquaporin-4-expressing (AQ) and -non-expressing (noAQ) cells, and demonstrated a clear difference between the signals from the two cell types. The data were interpreted using a two-compartment (intra and extracellular spaces) model including inter-compartmental exchange. It was also assumed that restricted diffusion of water molecules inside the cells leads to the intracellular diffusion coefficient being inversely proportional to the diffusion-time. Estimates of the water-exchange-times obtained with this model are compared to those measured using an independent optical imaging technique (coherent anti-Stokes Raman scattering imaging, CARS). For both techniques it was found that the exchange-time estimated for the noAQ cells was significantly longer than that for the AQ cells.

## Introduction

Cell membrane water permeability (CMWP) is altered in diseases like cancer^1,2^ and brain edema^3^. However, there is no medical imaging technique that can measure CMWP quantitatively. If such a technique were available for clinical use, it would prove very useful for disease diagnosis and therapy assessment.

An aquaporin (AQP) is a membrane channel protein that allows water molecules to be transported from one side of the membrane to the other. AQPs have been shown to regulate CMWP^4^. Recently, Ibata *et al*. reported a method to visualize water exchange between the intra- and extracellular spaces of AQP4 (a subtype of AQP) -expressing and -non-expressing cells using an optical imaging technique^5^. The technique they used is called coherent anti-Stokes Raman scattering (CARS) microscopy, which can selectively visualize H_2_O. After quick replacement of the extracellular H_2_O by D_2_O, they succeeded in measuring the exchange-time of water from the intracellular to the extracellular space of a single HeLa S3 cell (100.7 ms for AQP4-non-expressing cells and 43.1 ms for AQP4-expressing cells). From those measurements they were able to quantify the difference in CMWP for the two cell types after estimating the water exchange-time and cell size. Although it may be difficult to apply this method to an *in vivo* study, information about the CMWP of each cell type may be extremely useful for *in vitro* validation of a potential clinical CMWP imaging method.

Diffusion-weighted magnetic resonance imaging (DWI) plays an important role in the diagnosis of diseases such as brain infarction and cancer^6^. Although it is understood that signal attenuation is due to the diffusion of water molecules, a reliable quantitative signal model relating tissue parameters and signal contrast remains to be established. CMWP must affect the DWI signal^7^, but biological models used to interpret DWI usually treat the cell membrane as an impermeable wall^8–10^. One reason for this is that DWI is usually performed at diffusion-times (*Td*s) that are too short for the effect of CMWP on DWI signal to be easily observed. A second reason is that models that do account for CMWP are much more complicated. A third reason is that it is difficult to validate the measured quantitative CMWP value against a reliable standard. Several biological models that include cell permeability as a parameter of the system have been reported^11–15^, but direct validation of the measurements is difficult.

**Figure 1:**
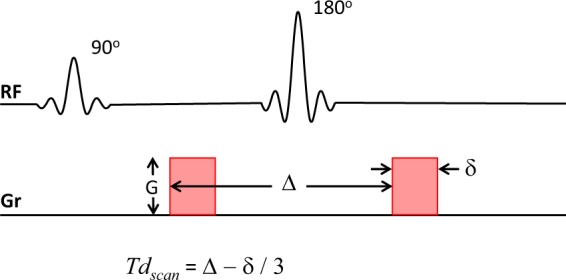
The PGSE sequence used to perform multi-b-value multi-*Td* (MbMTd) diffusion-weighted imaging (DWI). The sequence consists of 90° degree excitation and 180° refocusing RF pulses (RF) producing a spin echo, as well as magnetic field gradients (Gr) for diffusion weighting. The sequence diffusion-time (*Td*_*scan*_) is calculated using the separation of the diffusion gradient lobes (Δ) and diffusion gradient duration (δ) (*Td*_*scan*_ = Δ − δ/3). The *b*-value is the product of *Td*_*scan*_ with the square of the spatial frequency (*q*), where *q* = γGδ (γ, gyromagnetic ratio; G: gradient strength).

In this study we performed multi-b-value multi-*Td* (MbMTd) DWI on AQP4-expressing (AQ) and -non-expressing (noAQ) Chinese hamster ovary (CHO) cells over a relatively wide range of *Td*, which was modulated by setting the separation of the diffusion gradient lobes (Δ) to 40, 70, or 100 ms to change the sequence parameter *Td*_*scan*_ (Fig. 1). We also applied a simple DWI signal model that enabled us to estimate the exchange-time of water between the intra- and the extracellular spaces. The results were then compared with the results of the CARS experiments^5^.

## Theory

### Water exchange-time measurement with MRI

**Figure 2:**
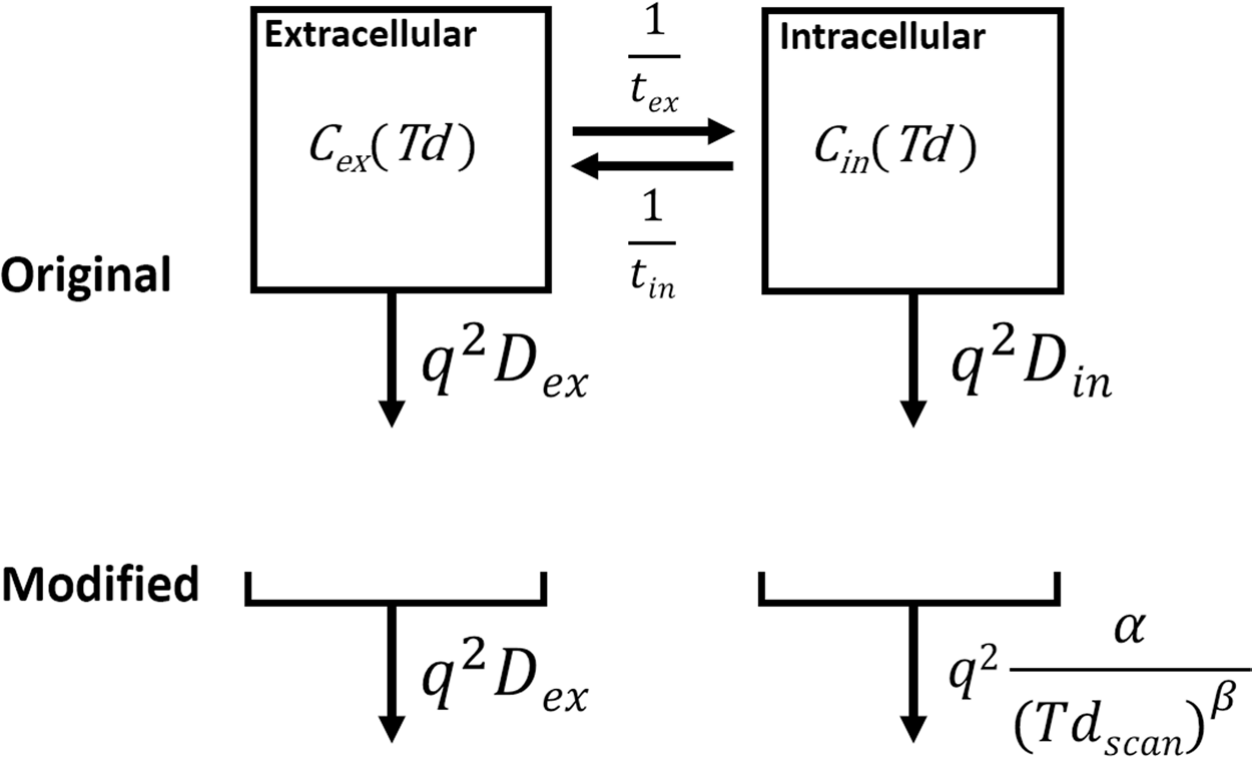
Two-compartment models including inter-compartmental exchange. *C*_*ex*_(*Td*) and *C*_*in*_(*Td*) are the normalized extracellular and intracellular signals, respectively, at diffusion-time *Td*. *t*_*ex*_ and *t*_*in*_ are constants representing the inter-compartmental lifetimes. *D*_*ex*_ and *D*_*in*_ are the diffusion coefficients in the extracellular and intracellular spaces, respectively. The water signal from each compartment decreases with a rate constant, *q*^2^*D*. In the original Kärger model (top), *D*_*ex*_ and *D*_*in*_ are both constant and *Td*-independent, while for the modified model (bottom) *D*_*ex*_ is constant, and *D*_*in*_ is modelled as “α/(*Td*_*scan*_)^*β*^”, where *Td*_*scan*_ is the experimental diffusion-time, “*β*” is a parameter to test our assumption that *D*_*in*_ is inversely proportional to *Td*_*scan*_, and “*α*” is a fitting parameter with units of length squared if *β* = 1. “*q*” is the q-value determined by the parameters of the motion probing gradient.

The Kärger model^15–17^, which is a simple two-compartment model with inter-compartmental compound exchange, was used as a base model for analysis (“Original” in Fig. 2). The model assumes free Gaussian diffusion of water in each compartment (ie *D*_*ex*_ and *D*_*in*_ are constant), but many papers indicate that *in vivo* water diffusion is non-Gaussian^18,19^. We modified the model (“Modified” in Fig. 2) under the assumption that *Td*_*scan*_ is sufficiently long that the diffusion coefficient in the extracellular space (*D*_*ex*_) is approximately constant, while that in the intracellular space (*D*_*in*_) is inversely proportional to the diffusion-time (see Discussion and Methods sections)^18^. The data was then analyzed using Td-independent *D*_*ex*_ and Td-dependent *D*_*in*_ as below,

1 $${D}_{in}=\frac{\alpha }{T{d}_{scan}^{\beta }},$$

where α has dimensions of length squared in the case that *β* = 1, while *β* is a parameter inserted to test the assumption that *D*_*in*_ is inversely proportional to *Td*_*scan*_ (“Modified” in Fig. 2).

Using the modified model, the MRI-based estimate of the exchange-time (*τ*_*MRI*_) is^14^,

2 $${\tau }_{MRI}={F}_{ex}\cdot {t}_{in}={F}_{in}\cdot {t}_{ex},$$

where *F*_*ex*_ and *F*_*in*_ are the signal fractions of the extracellular and intracellular spaces, respectively, and *t*_*ex*_ and *t*_*in*_ are the lifetimes in the extracellular and intracellular spaces, respectively. Note that *F*_*ex*_ + *F*_*in*_ = 1.

### Water exchange-time measurement with CARS

**Figure 3:**
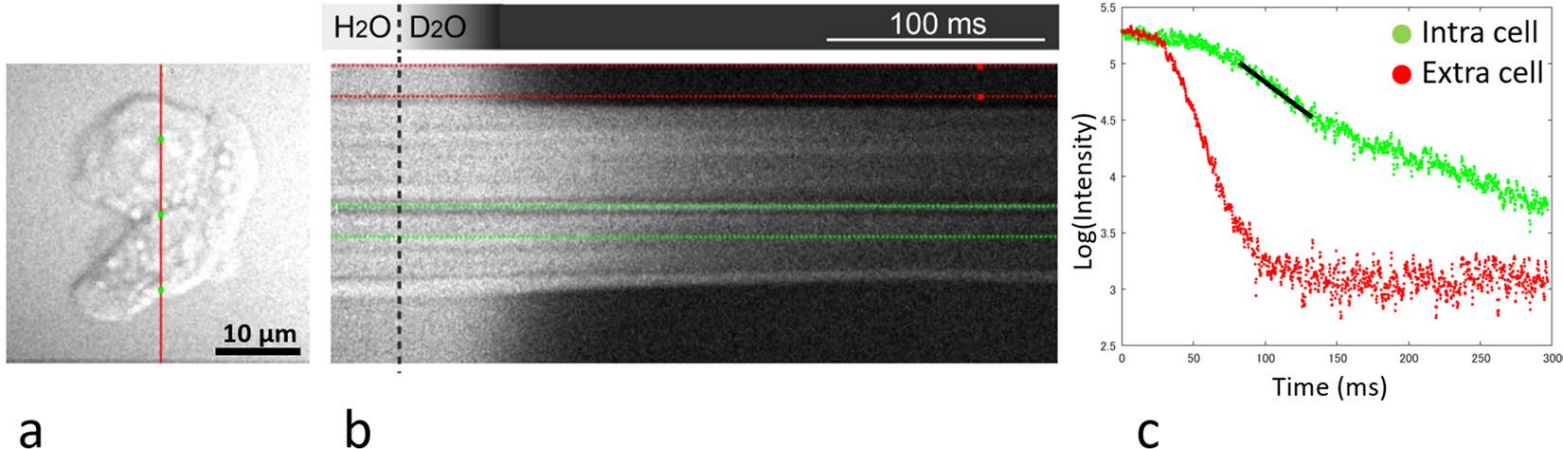
Coherent anti-Stokes Raman scattering (CARS) microscopy. This technique can selectively visualize H2O (**a**). After rapid replacement of H2O with D2O (no signal) in the extracellular space, water exchange between intracellular and extracellular space can be observed (**b**) using line scans taken where the red line in (**a**) is drawn. (**c**) is a logarithmic graph of the time-intensity curves for the intracellular (green dots) and extracellular (red dots) spaces. The plotted intensity is the mean value between the green or red colored lines in (**b**). Regression over a range of 50 ms was performed where the slope in (**c**) appears steepest (black line) on the logarithmic graph of the time-intensity curve, and the inverse of the slope from the fit was taken as the estimate of *τ*_*CARS*_.

The water exchange-times between the intracellular and extracellular spaces were also investigated with CARS microscopy. Ultra-high-speed line-scan CARS images were obtained every 0.488 ms. This technique can discriminate between H2O and D2O signal, as well as allow selective imaging of the extracellular and intracellular spaces. After rapid replacement of H2O with D2O in the extracellular space (replacement time is less than 20 ms), the intracellular H2O signal was observed. Details of the experiment can be found in the previous report by Ibata *et al*.^5^. The CARS-based estimate of the exchange-time (*τ*_*CARS*_) is calculated with

3 $${S}_{in}(t)=A{e}^{-\frac{t}{{\tau }_{CARS}}}+{\rm{B}}$$

where *S*_*in*_(t) is the signal intensity from inside a single cell, and A and B are constants. Regression over a range of 50 ms was performed where the slope appears steepest on a logarithmic graph of the time-intensity curve, and the inverse of the slope from the fit was taken as the estimate of *τ*_*CARS*_ (Fig. 3c).

## Results

### Multi-b-value multi-*Td* (MbMTd) DWI

As measured by an automated cell counter the survival rate of both the noAQ and AQ cells after MRI examination was more than 97%, and the radius of both cell types was in the range of 6.0–7.1 μm.

**Figure 4:**
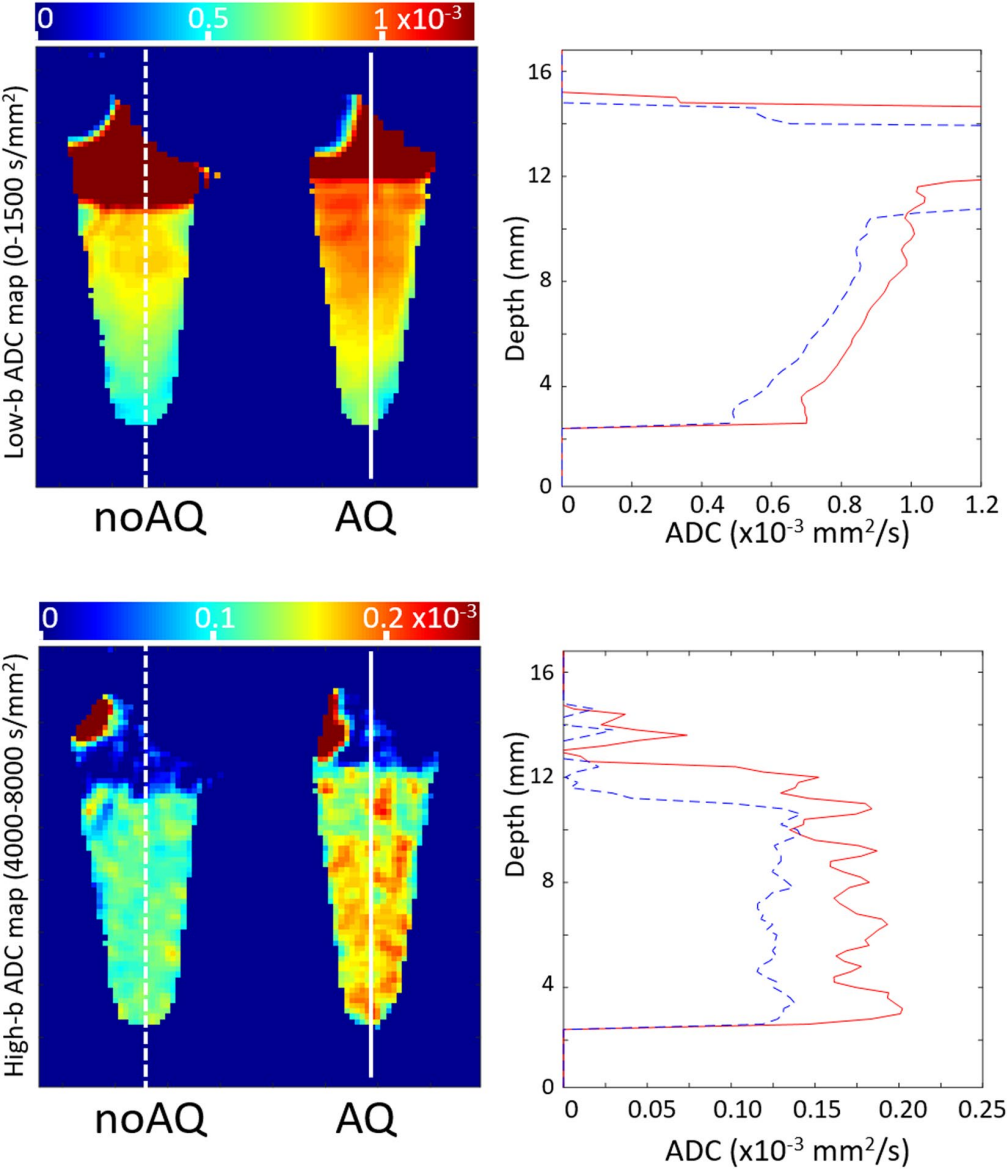
Profiles along lines drawn on ADC maps (×10^−3^ mm^2^/s) of aquaporin-4-expressing (AQ, solid lines) and -non-expressing cells (noAQ, dashed lines) in PCR tubes. The ADC maps were calculated from the Δ = 100 ms data, with the low-b and high-b ranges corresponding to b = 0–1500 s/mm^2^ and b = 4000–8000 s/mm^2^, respectively. The low-b ADC appears to depend on the depth within the sample tube, while the high-b ADC may be more sensitive to AQP4 expression.

**Figure 5:**
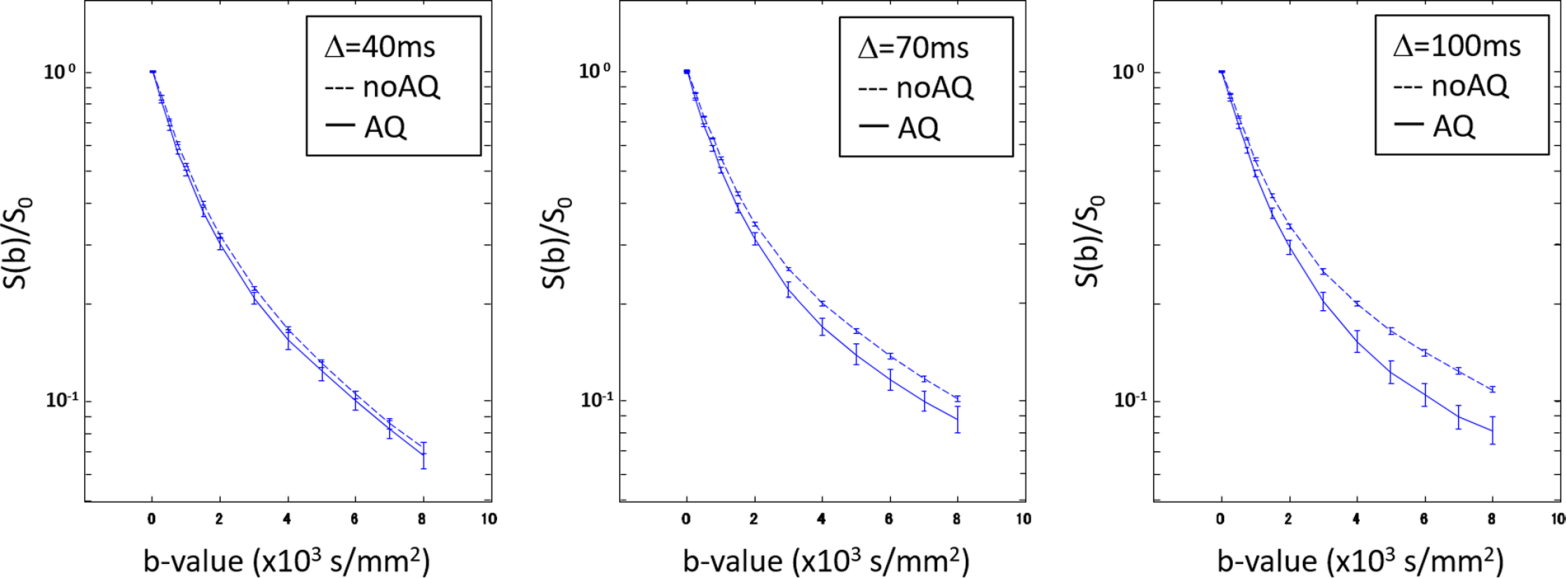
Normalized b-value-dependent signal decay for the noAQ and AQ samples at different diffusion-times. Separation between the curves of the two cell types increases with diffusion-time. Also of note is that the AQ signal at high b-value increases from Δ = 40 ms to 70 ms, but decreases from Δ = 70 ms to 100 ms. This behavior cannot be explained by a simple Gaussian or restricted diffusion model.

**Figure 6:**
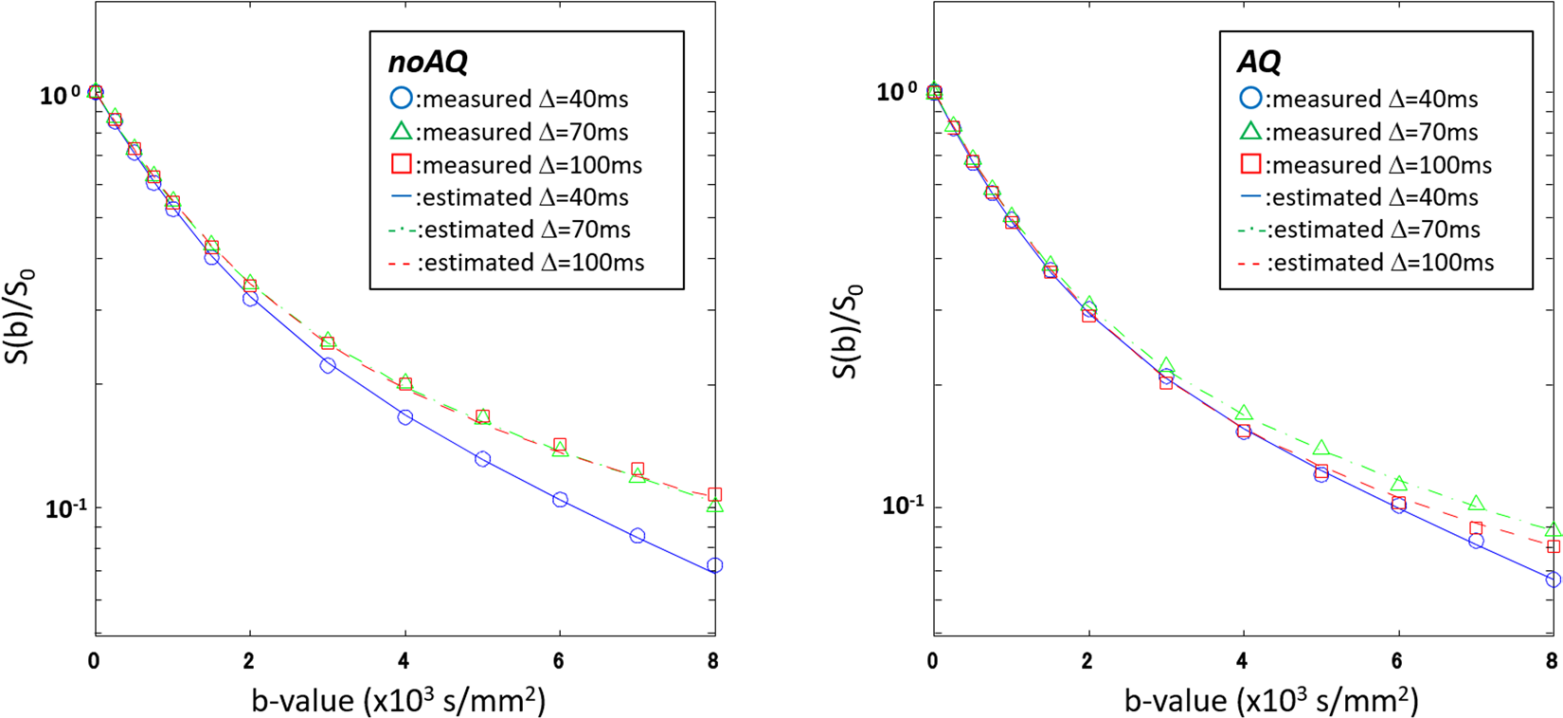
Curve fits using the proposed model. The graphs present fits to Eqs 7–9 for both the noAQ (left) and AQ (right) data using the proposed functional form: *D*_*in*_ = α/*Td*_*scan*_^*β*^ (“Modified” in Fig. 2). The fitted curves are consistent with the observed data for all *Td*s. All parameter estimates for the model are summarized in Table 1.

Separate apparent diffusion coefficient (ADC) maps were calculated for the images with b = 0–1500 s/mm^2^ (low b) and b = 4000–8000 s/mm^2^ (high b) ranges using single-exponential fitting to the data acquired with Δ = 100 ms (Fig. 4). The low-b range ADC map appeared to depend on depth, while the high-b range ADC map was more sensitive to differences in AQP4 expression. The mean b-value dependent signal changes from ROIs drawn midway down the cell cultures were compared in Fig. 5. The data were normalized by measurements made with b = 0. For the data measured at Δ = 40 ms, the noAQ signal was similar to the AQ signal across the full range of b values. At Δ = 70 ms both the noAQ and AQ signals decayed more slowly with b-value than at Δ = 40 ms. However, as the attenuation for noAQ was slower, there was a clear difference between the noAQ and AQ signals. The difference between noAQ and AQ was further enhanced as Δ was increased to 100 ms. The noAQ signal at Δ = 100 ms was similar to that at Δ = 70 ms, but that of AQ was slightly reduced from Δ = 70 ms to 100 ms, which is the primary source of the enhancement (Fig. 6).

### Model fitting

**Figure 7:**
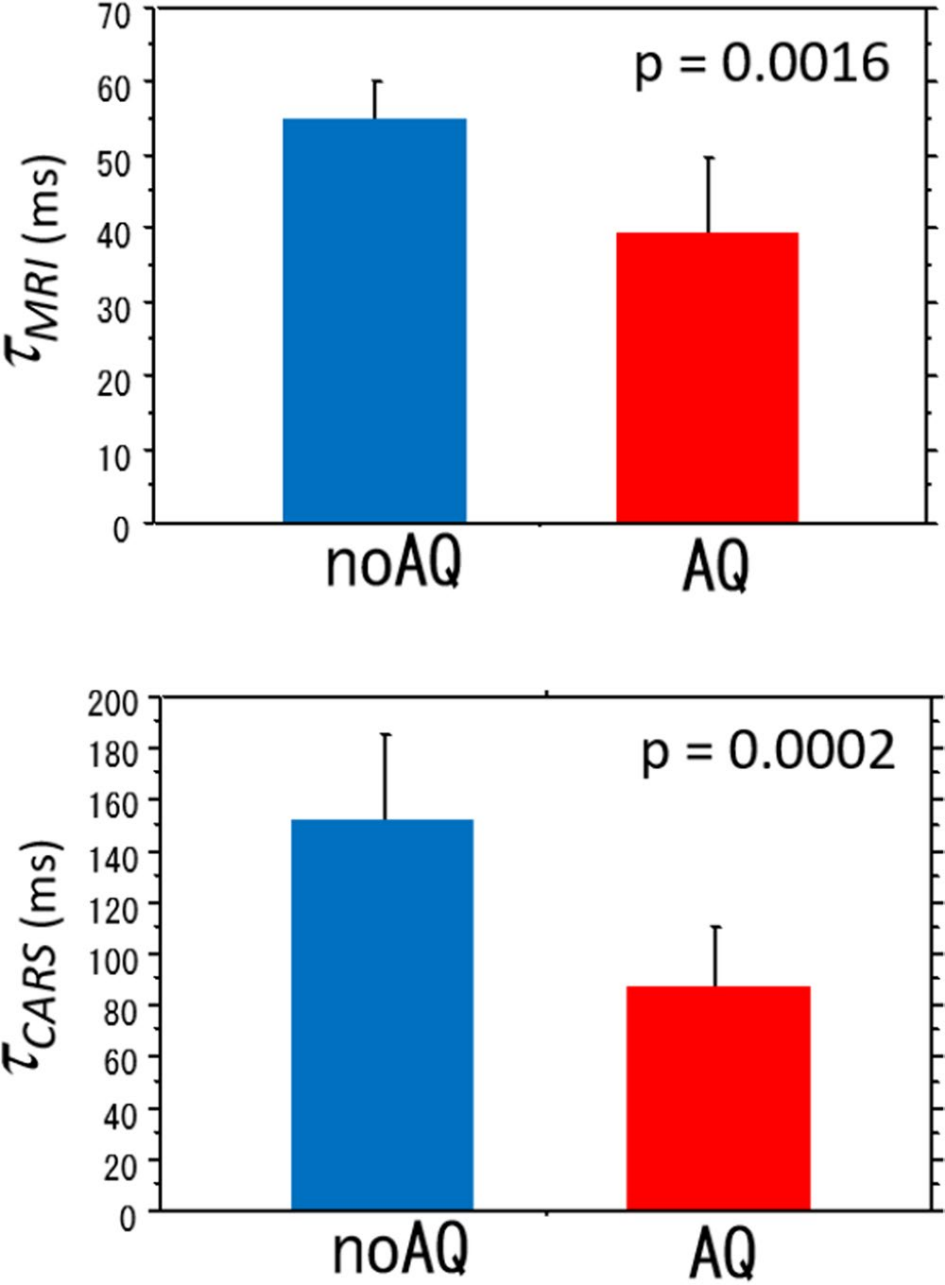
Exchange-times for AQP4-non-expressing (noAQ) and expressing (AQ) cells measured with MRI (τ_MRI_) and CARS (τ_CARS_). Both τ_MRI_ and τ_CARS_ for noAQ are longer than those for AQ. There are significant differences in both exchange-times between noAQ and AQ.

After fitting the data to the proposed model (“Modified” in Fig. 2), it was found that the fitted curves are consistent with the observed data across all Δs (Fig. 6). The estimates of *τ*_*MRI*_ for the noAQ and AQ cells were 55.1 ± 5.0 ms and 39.2 ± 10.3 ms, respectively (Fig. 7), and there was a significant difference between these two values (unpaired-t test, p = 0.0016). There was also a significant difference in the estimates of *t*_*in*_ for the two cell types (unpaired-t test, p < 0.0001). Other parameter estimates had no significant differences. The parameters *α* and *β* for the intracellular diffusion were 6.26 ± 0.10 μm^2^ and 0.955 ± 0.016 for noAQ, and 6.49 ± 0.44 μm^2^ and 0.968 ± 0.014 for AQ, respectively. All parameter estimates are summarized in Table 1.

**Table 1:** Estimates of the parameters for the “modified” two-compartment model with inter-compartmental exchange.

	t_in_(ms)	t_ex_(ms)	F_in_	D_ex_(×10^−3^ mm^2^/s)	α	β
noAQ	89.5 ± 5.4*	144 ± 21	0.385 ± 0.029	1.18 ± 0.08	6.26 ± 0.10	0.955 ± 0.016
AQ	61.1 ± 12.3*	112 ± 42	0.365 ± 0.044	1.35 ± 0.25	6.49 ± 0.44	0.968 ± 0.014

*t*_*in*_ and *t*_*ex*_, lifetimes in the intra- and extracellular spaces, respectively; *F*_*in*_, signal fraction from intracellular space; *D*_*ex*_, apparent diffusion coefficient for the extracellular space; *α* and *β*, parameters for the functional form *D*_*in*_ = *α/Td*_*scan*_^*β*^ for “Modified” in Fig. 2. * indicates a significant difference between the AQ and noAQ estimates (unpaired-t, p < 0.0001).

The exchange-time for individual cells of each cell type were also measured with the CARS technique (τ_CARS_). There was a clear significant difference in τ_CARS_ between the noAQ (152 ± 33) and AQ cells (87.7 ± 22.9 ms) (unpaired-t test, p = 0.0002, Fig. 7).

## Discussion

### Multi-b-value multi-*Td* (MbMTd) DWI

In this study, the low-b ADC map appears to depend on the depth within the samples, while the high-b ADC is more sensitive to depth-independent factors such as AQP4 expression (Fig. 4). It has been argued in a previous study that for the low-b value range, ADC mainly reflects the balance between extracellular and intracellular volume fractions^20^, which may be the main reason why the low-b ADC is more depth-dependent. The intracellular fraction of both cell types is expected to be larger at greater depth because deep cells are more densely packed after being centrifuged. On the other hand, at high b-value the signal may be more heavily weighted by the intracellular contribution because the motion of extracellular molecules is less restricted, and therefore the extracellular signal is more strongly attenuated with higher b-value than intracellular signal (extracellular signal at b = 4000 s/mm^2^ is less than 5% of the total signal)^21^. In this case it is likely that the remaining signal reflects factors that influence the diffusion of intracellular water molecules. Among these factors, the major physical difference between the two cell types is the AQP4 expression, which suggests that the different ADCs of the cell types at high b-value is related to CMWP.

### Model fitting

In this study we found a difference in the b-value-dependence of the normalized signal changes between noAQ and AQ cells. The difference in the signal was more noticeable at higher b-value and longer *Td*. The results are consistent with the concept that water has more time to exchange between extra- and intracellular spaces at longer *Td*. Therefore, we proposed a suitable model with which CMWP can be estimated from the *Td*-dependent signal changes.

As mentioned in the Theory section, the simplest model for DWI signal attenuation that includes inter-compartmental exchange is that reported by Andrasko and Kärger (Original in Fig. 2)^15–17^. This model assumes free Gaussian diffusion of water, in which case the diffusion coefficient of the water is independent of *Td*. Under this model, if the water exchange is zero, then the b-value-dependent signal decay curves should be similar for all *Td*s. In contrast, if the inter-compartmental water exchange is nonzero, the normalized b-value-dependent signal should decay faster at longer *Td*. However, the signal decay for both the noAQ and AQ cells at Δ = 70 ms was slower than that at Δ = 40 ms (Fig. 5), which means that the results are not consistent with this model. Fieremans *et al*. also suggested that the original Kärger model overestimates the exchange-time for cells with high permeability^14^.

As the original Kärger model is not suitable for describing *in vivo* water diffusion, we modified the model with consideration of the nature of intracellular diffusion. It has been suggested that intracellular diffusion might be roughly modeled as restricted diffusion inside a sphere of diameter *A*^22^. In that case, for long diffusion-times (*Td*» *A*^2^/2*D*; *D* is the free-water diffusion coefficient), the apparent diffusion coefficient is approximately inversely proportional to *Td*. Hence, the functional form adopted for *D*_*in*_ in Eq. 1. For the case of extracellular water diffusion, even though water molecules are hindered by the cell membrane to some extent, they may still travel very large distances in comparison to the cell dimension^8^. According to previous studies, the *Td*s used in the present work (37.7, 67.7, and 97.7 ms) are long enough to treat the observed *D*_*ex*_s as *Td*-independent^9,10,23,24^. Our previous work was also consistent with those reports (Supplementary Fig. S1)^25^. In accordance with these ideas, we analyzed the data using a *Td*-independent *D*_*ex*_ and the functional form α/*Td*_*scan*_^*β*^ for *D*_*in*_ (Modified in Fig. 2). As mentioned in the theory section, the parameter *α* has the dimensions of length squared in the case that *β* = 1, while *β* is a parameter inserted to test the assumption that *D*_*in*_ is inversely proportional to *Td*_*scan*_. The curve fitting matched the data well as shown in the Results section. In this fitting, the values of *β* were near 1 (0.955 ± 0.016 for noAQ, and 0.968 ± 0.014 for AQ), which is consistent with our assumption for the model. If *β* is equal to 1, α should be A^2^/5^18^, and the radii of the noAQ and AQ cells can be estimated as 5.7 and 5.8 μm, respectively. These values are consistent with those obtained from direct measurement (6–7.1 μm). Overall, our model works well for the analyses of DWI for the noAQ and AQ cell samples.

### Discrepancy in the exchange-times measured with MRI and CARS

Both τ_MRI_ and τ_CARS_ were longer for the noAQ cells, however, the former (88.9 ms for noAQ and 62.7 ms forAQ) are lower than those of the latter (152 ms for noAQ and 87.7 ms for AQ). There are a number of possible reasons for this. First, MRI does not visualize all water protons because some protons (e.g. bound water protons) have a T2 that is too short. Such water protons may have very long exchange-time. It follows that τ_MRI_ may be shorter than the exchange-time for all water molecules that is measured by CARS. Second, the replacement of extracellular H_2_O with D_2_O in the CARS experiment was very quick, but the exchange-time is nonzero (≈16.1 ms^5^). This exchange-time should be considered when assessing the true exchange-time. The difference in the exchange-time between MRI and CARS measurements for the AQ cells is small enough that it might be compensated in this way. However, the difference between τ_MRI_ and τ_CARS_ for noAQ cells is too large to be explained by this mechanism. Third, the range of the Td (37.7–97.7 ms) may be too low for accurate calculation of the exchange-time for noAQ cells. The CARS estimate of the intracellular water exchange-time for noAQ cells was 152 ms. Ideally, measurements should be performed over a diffusion-time range that includes this value. Fourth, even though the room temperature was kept at 22 C during experiment, it is possible that the temperature in the PCR tube increased due to RF and magnetic gradient switching. Ibata *et al*. reported that intracellular water exchange-time is shorter at higher temperature, and this is especially true for the noAQ cells^5^. Such a temperature difference may produce a large difference in the water exchange-time measured for noAQ cells with MRI and CARS. Finally, the difference in cell density between the samples used for MRI and CARS measurements may affect the results of the measured exchange-times. The cells were packed tight in the MRI sample, while the density was much lower in the CARS sample.

Although a difference in τ_MRI_ between the noAQ and AQ cells was successfully observed with our MRI technique, the quantitative values produced by the method still need to be validated in further studies. Similar experiments were performed by Thelwall *et al*. using human erythrocyte ghosts^11^. The reported exchange-time (20–24 ms) is smaller than those we obtained. The main reason for the difference with our measurements may be the difference in cell volumes; cells with the same permeability but larger volume will have longer exchange-time. Differences in the temperature condition during the performance of the experiments is another possible reason for the difference^12^.

### Potential of this method for human application

It is often difficult to observe Td-dependent ADC changes in normal tissue, but some reports suggest that pathological tissue, such as in the case of cancer^26^ and stroke^27^, may show characteristically large signal changes in DWI when Td is changed. Many clinical researchers are therefore interested in multi-Td measurements.

One advantage of our method is that it can easily be applied in clinical examinations. Since the *Td*_*scan*_s are relatively long, the magnetic gradient amplitude necessary to create high b-values is not too strong even for clinical systems. One difficulty is that a relatively long TE has to be used to achieve the long *Td*_*scan*_*s*, and consequently the signal-to-noise ratio (SNR) is lower than for usual clinical DWI. However, since the main targets of permeability measurement are white matter dysfunction and cancer, which have low ADCs, sufficient SNR will be available in those areas even when using long TE. Reports of the exchange-times in highly-permeable lesions, such as brain infarction and viable cancer, suggest that the diffusion-times available to our spin-echo method are sufficient for reliable quantitative measurements^5,27,28^. A stimulated-echo sequence (STE) is another possible way to lengthen *Td*^29^, although the SNR is only about 50% of spin-echo imaging. It should be remembered, however, that the longer mixing time (time between the 2^nd^ and 3^rd^ 90 degree pulses) required for longer Td may change T1-related signal contrast in STE DWI.

A survey of the literature finds that non-invasive clinical CMWP measurement has not yet been established. Although clinical MR scanners have more limitations than animal ones, clinical application of our method may provide new information concerning cell characteristics. Ozarslan *et al*. reported the diagnostic potential of diffusion-time-dependent MR signal changes^30^. AQP4 is known to express on the membrane of the endfeet of astrocytes, where it regulates permeability. Some reports have suggested that AQP4 expression affects the severity of brain infarction^2^. Measurement of AQP4 antibodies is used for clinical diagnosis of neuromyelitis optica (NMO), which may also be related to the function of AQP. Other types of aquaporin are also involved in diseases such as cancer, nephrogenic diabetes insipidus, and cataracts^31–33^. Therefore, the proposed method may be useful for the diagnosis of disease and therapy assessment.

## Limitations

A limitation of the model used in this paper is that it does not account for possible differences between intracellular and extracellular T1 & T2. Significant differences between the intracellular and extracellular relaxation times could lead to systematic errors in the parameter estimates, which could also be another source of discrepancy between the exchange-times measured with MRI and CARS. Several studies on intracellular and extracellular relaxation times have been published^34–36^. Proton relaxation in a biological environment is complicated; it involves cross-relaxation and chemical-exchange between protein and bound and free water protons, as well as water exchange between intra- and extracellular compartments. Nevertheless, there is no well-established model for intercompartment water exchange that includes both diffusion and T1 & T2 relaxation effects^13,27,37,38^. The possible influence of T1 and T2 relaxation should be considered more closely in future research.

Another possible limitation of the model is that it does not consider anisotropy of water diffusion, so it cannot simply be applied to water diffusion in white matter. Myelinated neuron fibers have such a small permeability that impermeable anisotropic models may be more useful^8,9^. Additionally, it should be noted that DWI at high-b values has a low signal-to-noise ratio, which means that the data should be analyzed carefully^39,40^. The noise signal floor in the experiments was at most 1% of the signal in the b = 0 images, which is much lower than the signal for the highest b-value.

## Conclusion

In conclusion, we performed multi-b and multi-diffusion-time DWI on AQP4-expressing and -non-expressing cells, and demonstrated a clear difference between the signals from the two cell types. The data were interpreted with a two-compartment model including inter-compartmental exchange, and this was used to estimate the water-exchange-time, which was consistent with one-cell measurement data obtained with CARS. The results indicate that this method might be used to characterize cell-membrane water permeability.

## Methods

### Cell culture and transfection

The cellular experiments in this study were performed in accordance with our local guidelines.

Chinese hamster ovary (CHO) cells stably transfected with either the expression vector pIRES2-EGFP, in which a unique AflII site had been modified to an EcoRI by linker ligation containing mAQP4 M1 cDNA (AQ), or the empty vector (noAQ) were maintained in Ham’s F-12 (Wako, Japan) supplemented with 10% FBS (fetal bovine serum) (Sigma), 1% penicillin/streptomycin (Life Technologies) and 0.5 mg/mL G418 antibiotics (Nacalai tesque) in 10-cm culture dishes at 37 °C in a humidified atmosphere (5% CO_2_–95% air). Stable CHO-cell clones were established as described in previous studies^41,42^. In brief, the plasmids were linearized with EcoRI and transfected into CHO cells with Lipofectamine and Plus reagents (Life Technologies). Forty-eight hours after transfection, cells were re-seeded onto ten 10-cm dishes and selected for ten days with G418. Several colonies showing green fluorescence were picked up and amplified.

### Cell suspensions

The cells were centrifuged at 800 rpm for 5 min at 4 °C. A suspension of 0.2 ml containing 2.5 × 10^7^ cells was prepared with PBS in a PCR tube (0.5 ml in size) for each cell type. Room temperature was maintained at 23 °C. A sample was extracted from each cell suspension prior to MR acquisition, and another was extracted after MR acquisition to detect possible changes over time. All samples were photographed using an inverted light microscope at a magnification of ×200. The percentages of cell survival were calculated with trypan blue, and the mean and the range of cell size was calculated from each photograph by an automated cell counter (Invitrogen Countess, Thermo Fisher Scientific, Japan).

### MRI acquisition

A 7T animal MRI (Kobelco with Bruker BioSpin, Japan) with a volume resonator for transmission (Bruker Biospin, Germany) and a quadrature surface coil for reception (mouse brain coil, Rapid biomedical, Germany) was used for this experiment. The two cell samples (noAQ and AQ) were set in the gantry on a PCR tube holder (homemade) together with a PBS sample as a reference. The temperature of the air around the sample was maintained at approximately 22 °C.

Multi-b-value multi-*Td* (MbMTd) DWI was obtained using a pulsed-gradient spin-echo (PGSE) sequence with four-shot EPI acquisition (TR = 3 s, TE = 115 ms, matrix size = 128 × 128, spatial resolution = 0.02 × 0.02 mm^2^, slice thickness = 2 mm). The separation of the diffusion-gradient lobes (Δ) was set at 40, 70, and 100 ms to change *Td*_*scan*_ while keeping TE constant. The diffusion-gradient duration (δ) was fixed at 7 ms for all experiments. For each *Td*_*scan*_, the b-value was increased from 0 to 8000 s/mm^2^ in 14 steps (0, 2, 250, 500, 750, 1000, 1500, 2000, 3000, 4000, 5000, 6000, 7000, and 8000 s/mm^2^) by increasing the gradient amplitude. The multi-b-value DWI scan time for each *Td*_*scan*_ was 3 min, which means that it took about 10 min for one set of MbMTd DWI. To check scan stability, 5 sets of MbMTd DWI were analyzed in this study.

### Model detail: Two-compartment models with inter-compartmental exchange

The Andrasko-Kärger model is a simple two-compartment model with inter-compartmental compound exchange (Fig. 6, Step 1), and is generally used for pharmacokinetic analyses^15–17^. The water signal (*C(Td)*) from each compartment decreases with a rate constant, *q*^2^*D*, where *q* is a spatial frequency determined by the parameters of the motion-probing gradient, and *D* is the diffusion coefficient of water. The constant *t*_*ex*_ represents the lifetime of water molecules in extracellular space before transferring to intracellular space, and *t*_*in*_ is the lifetime in the opposite direction. The extracellular and intracellular signals, *C*_*ex*_*(Td)* and *C*_*in*_*(Td)* evolve as a function of diffusion-time *Td* according to the following differential equations,

4 $$\frac{d{C}_{ex}(Td)}{dTd}=-\,({q}^{2}{D}_{ex}+\frac{1}{{t}_{ei}}){C}_{ex}(Td)+\frac{1}{{t}_{ie}}{C}_{in}(Td)$$

5 $$\frac{d{C}_{in}(Td)}{dTd}=-\,({q}^{2}{D}_{in}+\frac{1}{{t}_{ie}}){C}_{in}(Td)+\frac{1}{{t}_{ei}}{C}_{ex}(Td).$$

*D*_*ex*_ and *D*_*in*_ are the diffusion coefficients in the extracellular and intracellular spaces, respectively. *C*_*ex*_(*0*) and *C*_*in*_(*0*) are equal to the signal fractions of the extracellular (*F*_*ex*_) and intracellular (*F*_*in*_) spaces, respectively (note that *F*_*ex*_ + *F*_*in*_ = 1). Because water exchange from the extra- to intracellular space is equal to that from intra- to extracellular space, *F*_*ex*_ and *F*_*in*_ are related to the compartment lifetimes as follows:

6 $$\frac{{F}_{ex}}{{t}_{ex}}=\frac{{F}_{in}}{{t}_{in}}.$$

The total DWI signal is the *Td*-dependent sum of the extra- and intracellular concentrations,

7 $$\frac{S(Td)}{{S}_{0}}={C}_{ex}(Td)+{C}_{in}(Td),$$

where S0 is the signal for b = 0. After substituting the solutions of Eqs 4 and 5 into Eq. 7

8 $$\frac{S(Td)}{{S}_{0}}=(1-{F}_{in}^{\ast }){e}^{-{D}_{ex}^{\ast }{q}^{2}Td}+{F}_{in}^{\ast }{e}^{-{D}_{in}^{\ast }{q}^{2}Td},$$

9 $${D}_{ex}^{\ast },{D}_{in}^{\ast }=\frac{{D}_{ex}+{D}_{in}+\frac{\frac{1}{{t}_{ex}}+\frac{1}{{t}_{in}}}{{q}^{2}}\pm {({[-{D}_{ex}+{D}_{in}+\frac{-\frac{1}{{t}_{ex}}+\frac{1}{{t}_{in}}}{{q}^{2}}]}^{2}+\frac{4}{{q}^{4}{t}_{ex}{t}_{in}})}^{\frac{1}{2}}}{2}$$

and

10 $${{F}_{in}}^{\ast }=\frac{{F}_{ex}{D}_{ex}+{F}_{in}{D}_{in}-{{D}_{ex}}^{\ast }}{{{D}_{in}}^{\ast }-{{D}_{ex}}^{\ast }}.$$

Note that during fitting *q*^2^ is replaced by *b*/*Td*_*scan*_ to accommodate the direct dependence on b-value. *Td*_*scan*_ is a constant equal to the *Td* in each DWI scan. The data observed at each *Td*_*scan*_ was fitted to Eq. 8 as a function of b-value.

The model was then modified under the assumption that *Td*_*scan*_ is sufficiently long that the diffusion coefficient in the extracellular space (*D*_*ex*_) is approximately constant, while that in the intracellular space (*D*_*in*_) is inversely proportional to the diffusion-time. The data was then analyzed using a *Td*-independent *D*_*ex*_ and α/*Td*_*scan*_^β^ instead of a constant *D*_*in*_, where β is a parameter inserted to test the assumption that *D*_*in*_ is inversely proportional to *Td*_*scan*_ (“Modified” in Fig. 2), and the parameter α has dimensions of length squared in the case that *β* = 1.

Curve fitting was performed with the nonlinear least-squares method using Matlab (The MathWorks, Inc.). The method “lsqnonlin” in the Optimization Toolbox was used with the algorithm ‘trust-region-reflective’.

## Electronic supplementary material

Supplementary Information

## Data Availability

The datasets analyzed during the current study are available from the corresponding author on reasonable request.
