# Supplementary material for: Comparison of diffusion-weighted MRI and anti-Stokes Raman scattering (CARS) measurements of the inter-compartmental exchange-time of water in expression-controlled aquaporin-4 cells
Source: Sci Rep. 2018 Dec 18;8:17954. doi: 10.1038/s41598-018-36264-9 (PMC6298983; doi:10.1038/s41598-018-36264-9)
Supplement: Supplementary file 1 — Supplementary Information [file 41598_2018_36264_MOESM1_ESM.docx]

Supplementary Information

**Comparison of diffusion-weighted MRI and anti-Stokes Raman scattering (CARS) measurements of the inter-compartmental exchange-time of water in expression-controlled aquaporin-4 cells.**

Takayuki Obata^1,^ *, Jeff Kershaw^1^, Yasuhiko Tachibana^1^, Takayuki Miyauchi^2,3^, Yoichiro Abe^2,3^, Sayaka Shibata^4^, Hiroshi Kawaguchi^5^, Yoko Ikoma^1^, Hiroyuki Takuwa^6^, Ichio Aoki^4^, and Masato Yasui^2,3^

1. Applied MRI Research, National Institute of Radiological Sciences, QST, Chiba, 263-8555, Japan
2. Department of Pharmacology, Keio University School of medicine, Tokyo, 160-0016, Japan
3. Keio Advanced Research Center for Water Biology and Medicine, Tokyo, 160-0016, Japan
4. Department of Molecular Imaging and Theranostics, National Institute of Radiological Sciences, QST, Chiba, 263-8555, Japan
5. Human Informatics Research Institute, National Institute of Advanced Industrial Science and Technology, Tsukuba, 305-8566, Japan
6. Department of Functional Brain Imaging Research, National Institute of Radiological Sciences, QST, Chiba, 263-8555, Japan


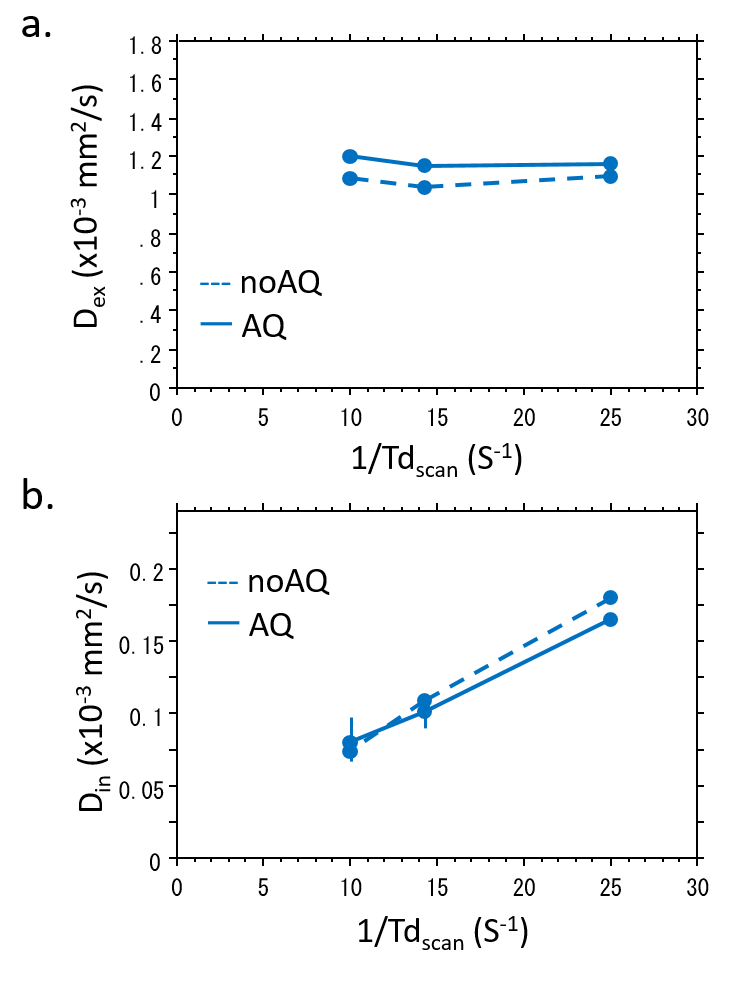


**Supplementary Fig. S1: *D_ex_* (a) and *D_in_* (b) at each Tdscan analyzed with the original Kärger model.**

Using the original K**ä**ger model (Original in Fig. 2), we calculate *D_ex_* and Din at each *Td_scan_* (37.7 ms, 67.7 ms, or 97.7 ms). The estimates of *D_ex_* were insensitive to *Td_scan_* for both cell types (a). In contrast, the estimates of *D_in_* derived from AQ and noAQ data are both inversely proportional to *Td_scan_* (b). The results are also consistent with our assumption.

**Table S1**

**Abbreviations lists**

| Abbreviation | dimensions |  |
| --- | --- | --- |
| *AQP* |  | aquaporin |
| *noAQ and AQ cells* |  | non-AQP-4-expressing and AQP4-expressing cells |
| *CARS* |  | coherent anti-Stokes Raman scattering |
| *CMWP* |  | Cell membrane water permeability |
| *DWI* |  | Diffusion-weighted magnetic resonance imaging |
| *MbMTd* |  | multi-b-value multi-*Td* |
|  |  |  |
| *Td* | *s* | diffusion-time |
| *Td_scan_* | *s* | sequence diffusion-time |
| *Δ* | *s* | separation of the diffusion gradient lobes |
| *δ* | *s* | duration of each diffusion gradient lobe |
| *C_ex_(Td)* |  | normalized water signal from the extracellular space |
| *C_in_(Td)* |  | normalized water signal from the intracellular space |
| *F_ex_* |  | extracellular signal fraction, equal to *C_ex_(0)* |
| *F_in_* |  | intracellular signal fraction, equal to *C_in_(0)* |
| *ADC* | *m^2^/s* | apparent diffusion coefficient |
| *D_ex_* | *m^2^/s* | apparent diffusion coefficient in the extracellular space |
| *D_in_* | *m^2^/s* | apparent diffusion coefficient in the intracellular space |
| *q* | */m* | spatial frequency |
| *t_ex_* | *s* | lifetime in the extracelluar space |
| *t_in_* | *s* | lifetime in the intracelluar space |
| τ*_MRI_* | *s* | exchange-time estimated with MRI |
| τ*_CARS_* | *s* | exchange-time estimated with CARS |
